# Coupling Aqueous Phase Chemical Actinometry with EPR Spectroscopy: An Approach for Probing Photochemical Processes at the Air–Sea Interface

**DOI:** 10.1002/cphc.202500734

**Published:** 2026-02-10

**Authors:** Daniele Scheres Firak, Thomas Schaefer, Bochao Yang, Olenka Jibaja Valderrama, Manuela van Pinxteren, Hartmut Herrmann

**Affiliations:** ^1^ Atmospheric Chemistry Department (ACD) Leibniz Institute for Tropospheric Research (TROPOS) Leipzig Germany

**Keywords:** actinometer, protoporphyrin XI, spin‐probing, superoxide radical, TEMPOL

## Abstract

Coupling investigations of the photochemistry of ambient samples with the detection of photoproduced radicals is a field of ongoing interest. Herein, we investigated the use of chemical actinometers to account for the photon flux in in situ electron paramagnetic resonance (EPR) spectroscopy experiments (*I*_EPR_) and correct it for solar light exposure. The developed method is based on the production of singlet oxygen (^1^O_2_) and its detection in spin‐trapping with TEMP‐OH in the photosensitization of Protoporphyrin IX (PPIX) and rose bengal (RB). Similar *I*_EPR_ values were achieved in both PPIX and RB systems, with values ranging from (1.3 ± 0.2) × 10^−8^ ≤ *I*_EPR_ ≤ (1.6 ± 0.3) × 10^−8^ mol photons L^−1^ s^−1^. TEMP‐OH protonation was shown to interfere with the results at pH < 2. The aggregation effects of PPIX were investigated, and solutions were shown to be stabilized by the presence of TEMP‐OH. The photochemical activity of the sea‐surface microlayer (SML) samples was subsequently probed in in situ EPR experiments for the first time and corrected for the equivalent solar photochemical activity. The average results of the sunlight‐induced oxidant formation rate were (3.8 ± 0.5) 10^−8^ M s^−1^, demonstrating the pronounced photochemical activity present in SML samples.

## Graphical abstract

Photochemical investigations were coupled with radical detection for quantifying oxidative processes in sea‐surface microlayer (SML) samples. A chemical actinometry approach was employed to measure photon flux in in situ EPR experiments and under solar irradiation. A significant sunlight‐induced oxidant formation was demonstrated in the SML samples, revealing the pronounced photochemical reactivity of this interface.

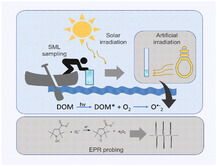

## 1 Introduction

Many natural processes are affected by sunlight irradiation, driving the conversion of organic and inorganic compounds from precursors to photoproducts in aqueous natural environments [1, 2]. Following these processes in situ is not always feasible due to the complexity of matrices and the instability of reaction products. Therefore, researchers typically appeal to offline analysis using simulated solar irradiation. This choice, despite simplifying the analysis routine, can be compromised by the lack of a proper comparison between the simulated environment and real environmental conditions. Accordingly, great care should be taken when designing photochemical laboratory experiments that mimic environmental processes.

Chemical actinometers are used to determine the photon flux density (*I*), the number of photons incident on a photochemical reactor per unit of area or volume under controlled conditions of light exposure, temperature, and volume [3, 4]. The estimation of *I* values enables accurate quantum yield determination, kinetic modeling, and ensures reproducibility in photochemical experiments [5, 6]. In artificial light photoreactors, potassium ferrioxalate and iodide‐iodate actinometers are commonly used, both relying on light‐induced redox reactions with high quantum yields in the UV range and effective between 200 and 500 nm [3]. In solar photochemistry, *I* measurements are complicated by inhomogeneous light distribution and cloud coverage. By selecting actinometers with adequate visible‐range quantum yields and considering cloud coverage, a reliable solar *I* can be obtained [5, 6]. Typical actinometers include valerophenone, p‐nitroanisole/pyridine, and nitrate/nitrite [5, 6, 7], with product analysis performed in UV–vis spectroscopy. Due to the challenges associated with solar photoreactors, the characterization of *I* and the target experiment are best performed simultaneously to minimize calibration errors [6].

Although the previously mentioned actinometers can be helpful to characterize photochemical reactors with larger volumes or in flow systems, they are not practical for experiments involving small volumes and confined reactors. In in situ electron paramagnetic resonance (EPR) spectroscopy analysis, samples are typically loaded into capillary tubes in volumes lower than 50 μL and placed inside EPR resonators for irradiation. EPR resonators can have frontal windows that allow for the incidence of light, thereby permitting the acquisition of EPR spectra simultaneously with irradiation experiments. However, the acquisition of UV‐spectra, required for most available actinometer experiments, poses a challenge. Therefore, methods based on the direct monitoring of EPR signals must be used. Herein, we investigated a method for *I* determination based on the formation of singlet oxygen (^1^O_2_) in the irradiation of porphyrin and dye solutions in the presence of the ^1^O_2_ spin‐trapping agent 4‐hydroxy‐2,2,6,6‐tetramethylpiperidin (TEMP‐OH) [8, 9]. Porphyrins and dyes act as photosensitizers, absorbing radiation to form triplet states that decay in reactions with molecular oxygen, yielding ^1^O_2_ [10, 11, 12]. The number of photons absorbed to yield ^1^O_2_ can be inferred by measuring the decay of dissolved oxygen concentrations or the formation of ^1^O_2_, and is called quantum yield for ^1^O_2_ production (Φ^1^O_2_). When the diamagnetic TEMP‐OH reacts with ^1^O_2_, the EPR‐active TEMPOL aminoxyl radical with a characteristic EPR spectrum is formed. This adduct can be used to identify and quantify the production of ^1^O_2_.

While adequately considering the wavelengths of solar radiation and the volumes used in EPR spectroscopy, the use of photosensitizers for the determination of quantum flux can introduce errors associated with the variability in the reported Φ^1^O_2_ values. Porphyrins and dyes can undergo aggregation and photobleaching, which typically reduces the Φ^1^O_2_ [11, 13, 14, 15, 16]. This effect is especially important in higher concentrations of aqueous photosensitizer solutions [11]. The choice of pH and solvent has also been shown to affect the measured values of Φ^1^O_2_. Apart from aggregation effects, the solvent can substantially change the measured Φ^1^O_2_ values by affecting the half‐life of ^1^O_2_, which decays back to triplet oxygen via nonradiative energy transfer to the surrounding solvent molecules in the form of vibrational energy [17]. The half‐life of ^1^O_2_ is typically lower in water, with measured values around 2 μs, and higher in aprotic solvents, reaching 700 μs in CCl_4_ [18]. Therefore, experiments employing photosensitizers must be conducted in similar conditions as those used for Φ^1^O_2_ determination, and attention should be paid to the concentration of aqueous solutions and to aggregation effects.

The present study aims to characterize and optimize a method for estimating *I* values during in situ EPR experiments and solar light irradiation, and further apply this approach to a natural system as a proof of concept. To demonstrate the applicability of this approach beyond controlled systems, it was tested on sea‐surface microlayer (SML) samples, which represent a natural, highly complex interface directly exposed to solar radiation. The irradiation of SML samples is particularly suitable as a proof of concept, since the SML is often enriched with organic matter and trace metals that make it both photochemically active and representative of environmentally important processes at the air‐sea boundary [19]. Typically, SML samples contain high concentrations of dissolved organic matter (DOM), fluorescent‐DOM, and colored DOM [20, 21, 22, 23], which act as photosensitizers for the formation of reactive oxygen species (ROS), such as singlet oxygen and superoxide radicals (O_2_^−^), during the solar irradiation of the SML [24, 25]. Superoxide radicals can form hydrogen peroxide and trigger photo‐Fenton reactions in the presence of trace dissolved metals [24, 25].

The investigation of ROS is frequently accomplished in EPR spectroscopy with the use of spin‐trapping agents and probes [26, 27, 28]. Hydroxylamines are recognized probes for the investigation of O_2_^−^ radicals due to their higher stability and reactivity as compared to conventional nitroxide spin‐trapping agents [27]. Hence, the hydroxylamine 1‐hydroxy‐3‐methoxycarbonyl‐2,2,5,5‐tetramethylpyrrolidine (CMH) was here investigated as a radical probe for photoproduced ROS in SML samples. Several investigations have aimed to unravel the composition of SML samples and their conversion under light exposure, but an overall estimation of the photochemical activity using a simple and robust method has been lacking so far.

## 2 Experimental Section

### 2.1 In Situ EPR Settings

The in situ EPR determination of the quantum flux was investigated in a 50 μL capillary tube, filled to a volume of 10 μL and centered in a Bruker 4103TM resonator in a Bruker EMX Plus spectrometer. The Bruker 4103TM cavity is particularly suitable for the present investigation, as it can be used in the analysis of samples with high dielectric loss, features an optical window, and is designed for 80% transmission of incident light. The resonator was coupled with an optical fiber accessory preceded by a 1.0 mm SCHOTT WG280 filter and irradiated with a 150 W Xenon arc lamp (Hamamatsu Photonics). The capillary tubes containing the samples were made of glass, and the cut‐off wavelength of the system is shown in Figure S1. The EPR spectrometer operational settings were: microwave frequency 9.853 GHz, modulation amplitude 1.00 G, magnetic field scan 150 G, sweep time 15 s, conversion time 10 ms, time constant 5 ms, and two accumulations. The temperature of the resonator was controlled and kept at 298 K. For the kinetics experiments, spectra were acquired in the field delay mode at a 1 s scan delay. For signal intensity correction, a Cr^3+^ marker (*g* = 1.98, Bruker) was simultaneously measured with all samples. Spectral simulation and quantitative analysis were performed in the SpinCount software package of Xenon (Bruker Corporation). The accuracy of the system calibration was verified against the signal of 2,2,6,6‐tetramethyl‐1‐piperidinyloxyl (TEMPO) (99%, Sigma–Aldrich).

### 2.2 Photon Flux Determination

Two different actinometer solutions were used to characterize the photon flux of solar (*I*_SUN_) and in situ EPR (*I*_EPR_) experiments. Rose bengal (RB, CAS 632‐69−9, Sigma–Aldrich) solutions were used as a reference compound for singlet oxygen generation, with reported Φ^1^O_2_ in water varying from 0.75 to 0.76 [29]. Protoporphyrin IX (PPIX, CAS 553‐12−8, TargetMol) was used as a comparison, as reported Φ^1^O_2_ are less well‐defined.

Porphyrin solutions were prepared daily by weighing specific amounts of PPIX and dissolving it in a 0.5% NaOH solution. Subsequently, this solution was diluted to 10 μM and acidified with HClO_4_ to a pH range of 7.4–7.6. Daily spectra of 10 μM PPIX solutions were registered to guarantee a constant final concentration of porphyrin. Ethanolic and acidic solutions of PPIX in the same final concentrations were also prepared. RB solutions were prepared in water with a final pH ranging from 1 to 7.5 (adjusted with HClO_4_).

PPIX and RB solutions were added to capillary tubes in the presence of 20 mM of TEMP‐OH (4‐hydroxy‐2,2,6,6‐tetramethylpiperidin, CAS 2403‐88−5, Sigma–Aldrich). Concentrations of TEMP‐OH were optimized to achieve the best responses for RB and PPIX photolysis, simultaneously minimizing the production of radicals via photolysis of the spin‐trapping agent. Concentrations in the range of 2 mM showed no apparent signals, while at 200 mM, a high contribution of signals from TEMP‐OH photolysis was observed. The capillaries were centered in the EPR resonator and irradiated in situ, or placed on aluminum foil and irradiated during clean‐sky days in the solar peak hours. In experiments using solar irradiation, several capillary tubes were filled with the photosensitizer solution containing 20 mM TEMP‐OH. At predetermined times, the capillary tubes were removed from sunlight, wrapped in aluminum foil, and immediately analyzed in the EPR, using the same spectrometer settings as those employed in the in situ EPR experiments. The solar irradiance was simultaneously measured using a silicon photodiode pyranometer (Model: ML‐020VM) located on the rooftop of one of the buildings of the Leibniz Institute for Tropospheric Research (TROPOS), which is part of a larger pyranometer network built by the Remote Sensing Department of TROPOS [30]. The solar experiments were conducted on two different days (March 6–10, 2025), chosen according to their similar profiles of solar irradiance (Figure S4).

### 2.3 Photochemical Production of ROS in Sea‐Surface Microlayer Samples

SML samples were collected in a mesocosm experiment conducted at the Sea Surface Facility (SURF), at the Institute for Chemistry and Biology of the Marine Environment (ICBM) in Wilhelmshaven (Germany), between May 18th and June 16th, 2023. Samples were collected using the glass plate technique [21]. Additional information on the SML samples composition, the mesocosm experiment, and concentrations of specific organic components can be found in another publication [31] and in the Supporting Information file (Section 6). For the estimation of the total photochemical activity, the samples were added with 1 mM of the spin probe CMH (1‐hydroxy‐3‐methoxycarbonyl −2,2,5,5‐tetramethylpyrrolidine, NOXYGEN). Stock solutions of CMH were prepared daily at a concentration of 10 mM in deaerated ultra‐pure water. A blank of the CMH stock solution irradiated in ultra‐pure water was measured before each analysis. SML samples were transferred to a 50 μL capillary tube, centered in the resonator, and irradiated for 30 min. Control experiments with nonirradiated samples showed only a very minor radical signal over the monitored 30 min, likely attributable to interactions between trace metal ions and CMH. This background signal was markedly lower than the radical formation observed under photolysis conditions, indicating that the blanks do not significantly contribute to the measured photolysis rates of the probe molecule. In solar experiments, samples were added to the capillary tubes and exposed to sunlight on clear‐sky days at the solar peak hour. The pH of the samples was not modified during the analysis and was kept at 8.4 ± 0.1.

## 3 Results and Discussion

### 3.1 Method Development: Photon Flux Determination

In contrast to the well‐established Φ^1^O_2_ reported for aqueous solutions of RB, literature values of Φ^1^O_2_ for PPIX are scarce. In DMSO, a reported value of 0.6 is extracted from the paper of Gotardo et al. (2017) [32], which investigated the triplet state quantum yield (Φ_T_) of PPIX. The value considers the Φ^1^O_2_ as 0.79 × Φ_T_. A value of 0.77 was obtained from direct measurement of singlet oxygen in near‐infrared spectroscopy in phosphate buffer solutions (PBS) [33]. Values ranging from 0.54 to 0.6 are reported for aqueous solutions irradiated by an Xe–Hg lamp, measured at different wavelengths [34]. Table 1 summarizes the main reported Φ^1^O_2_ values for PPIX. The mechanism for ^1^O_2_ production via PPIX photosensitization is described in (I). PPIX absorbs photons to yield an excited triplet state PPIX*, which further reacts with dissolved molecular oxygen to yield ^1^O_2_, regenerating PPIX. The production of ^1^O_2_ is not the only pathway of energy transfer in photosensitization processes. A charge transfer mechanism can also occur, and in this case, superoxide radicals and PPIX radical cations are formed (II) [12]. The measured Φ^1^O_2_ values reflect the extent to which the channel described by (I) predominates over other decay channels [12, 35].

**TABLE 1:** Literature compilation of available values for the Φ^1^O_2_ measured in the irradiation of PPIX.

Φ^1^O_2_	Solvent/cPPIX	Irradiation source	*λ*, nm	Ref.
0.77	PBS^a^/TX100^b^ pH 7.4/8.3 μM	Coumarin/DCM^c^ laser	500/630	[33]
0.6	DMSO/50 mM	Nd:YAG^d^ laser	532	[32]
0.6	PBS/TX100 pH 7.4/10 μM	200 W Hg–Xe arc lamp	646	[34]
0.54	—	—	546/630	[34]

a PBS refers to phosphate buffer solution.

b TX100 refers to Triton X‐100.

c DCM refers to 2‐[2‐4‐(dimethylamino) phenyl]ethenyl]‐6‐methyl‐4H‐pyran‐4‐ylidene]‐propanedinitrile.

d Nd:YAG refers to Nd:YAG neodymium: yttrium aluminum garnet.



(I)

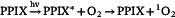

(II) $$\mathrm{PPIX}\;\overset{\mathrm{hv}}{\to }\;{\mathrm{PPIX}}^{*}+O_{2}\;\to \;{\mathrm{PPIX}}^{\cdot +}+O_{2}^{\cdot -}$$

Since most of the Φ^1^O_2_ values are available for aqueous buffered solutions, EPR experiments were conducted at pH 7.4–7.6. Control experiments showed that PBS substantially reduced the generation of TEMP‐OH adducts in the photosensitization of PPIX (data not shown). Therefore, solutions were prepared in NaOH and acidified with HClO_4_. Blanks of irradiated TEMP‐OH in the presence of NaOH solutions acidified to pH 7.5 with HClO_4_ showed no signals. Considering the reproducibility of the daily measured PPIX spectra, we considered the solutions to be stable under the investigated pH and PPIX concentrations.

Both RB and porphyrin solutions are prone to aggregation and formation of dimers and higher‐order structures [11, 13], which substantially affect the formation of triplet states and consequently the production of singlet oxygen. These effects can be ruled out or minimized in diluted solutions. Therefore, concentrations between 10 and 2 μM were used in the experiments, following recommendations from studies that investigated agglomeration effects in both systems [11, 13, 14].

**FIGURE 1:**
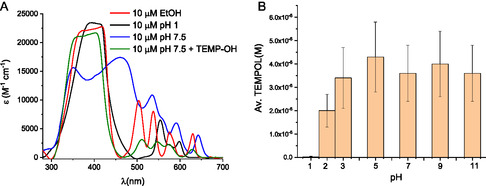
(A) Spectral analysis of PPIX solutions in different preparations. (B) The pH dependence of TEMPOL formation during irradiation of RB solutions.

The aggregation effects can be investigated in spectral analysis. Figure 1A displays the spectra of PPIX solutions in different preparations. A lower formation of dimers and higher‐order structures was achieved with dilutions of ethanolic and acidic PPIX solutions prepared in HClO_4_. In the spectra shown in Figure 1A, it is evident that the band centered at around 400 nm, known as the Soret band, is less broadened at pH 1 and in ethanolic solutions [14]. At pH 7.5, however, a broadening and a significant shift of the Soret band are observed, indicating a significant degree of aggregation. The determined molar absorption coefficient of 14,000 M^−1^ cm^−1^ at 400 nm and the spectral distribution indicate aggregation [13]. In general, aqueous porphyrin solutions prepared at pH 7.5 are always added with surfactants to suppress aggregation (as seen in Table 1). Here, we chose not to prepare PPIX solutions in the presence of surfactants, as those would compete with TEMP‐OH in reactions with formed radicals. Interestingly, in aqueous solutions prepared with 20 mM TEMP‐OH, the porphyrin spectrum exhibits different features, with fewer formed aggregates (green line shown in Figure 1A). This indicates TEMP‐OH acts as a stabilizer for aqueous solutions, promoting the formation of monomeric porphyrins and consequently maximizing the production of singlet oxygen at pH 7.5. It is the first time that the stabilization effect of spin trapping agents in aqueous solutions of porphyrins is reported. This effect can contribute to the application of EPR investigations of porphyrin solutions, especially relevant in the field of photodynamic therapy.

Although the aggregation of PPIX solutions was minimized at acidic pH, the EPR experiments at pH 1 resulted in no signals due to protonation effects of TEMP‐OH. Investigations of the protonation effects were conducted using RB solutions of 2 μM, considering that the Φ^1^O_2_ of RB solutions is pH‐independent. The formation of TEMPOL signals was smaller at pH below 2. From pH 3 to pH 11, the evolution and final concentration of radicals were equal amongst the different tested pH levels (Figure 1B). Protonation effects of TEMP‐OH and related piperidines can affect the observed yields of singlet oxygen [32]. The absence of signals at pH 1 and the lower concentrations at pH 2 indicate that protonation effects are critical at these pH levels for the used RB and PPIX concentrations.

Photobleaching can also decrease the ^1^O_2_ production in both PPIX and RB systems via reactions between the radicals formed during the photosensitizer irradiation and the photosensitizer structure [15]. In EPR experiments, the excess TEMP‐OH captures ^1^O_2_ and other formed radicals, minimizing the bleaching effect. Here, the evolution of singlet oxygen was monitored for 30–60 min, but only the linear phase of TEMPOL formation was considered for *I* estimations. Photobleaching effects might cause changes in the linearity. The stabilization of PPIX promoted by TEMP‐OH increases the absorption in the Soret band region and minimizes absorption in other spectral regions that can lead not only to a decrease in PPIX* but also to the promotion of other pathways for radical generation and PPIX photobleaching. The evolution of the spectra of PPIX aqueous solution in the absence of TEMP‐OH was monitored during irradiation experiments, and a significant effect of photobleaching was observed already within the first 15 min of irradiation (Figure S2).

**FIGURE 2:**
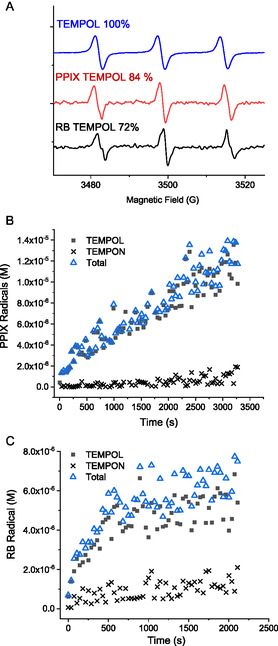
(A) TEMP‐OH adducts formed during the irradiation of PPIX and RB solutions. TEMPOL Δ*H*_pp_ = 1.6 G, aN = 16.99 G, and *g* = 2.00556. TEMPON Δ*H*_pp_ = 0.82 G, aN = 16.08 G, and *g* = 2.00549. Profile of TEMPOL and TEMPON formation during irradiation of (B) PPIX and (C) RB solutions.

The EPR spectra obtained for the in situ irradiation and generation of ^1^O_2_ via PPIX and RB photosensitization are shown in Figure 2A, and the chemical mechanisms involved in the formation of the different observed adducts are given in III and IV [36]. There is an obvious line split in the irradiation of RB, corresponding to the formation of TEMPON radical, the adduct formed in the reaction between TEMPOL and OH radicals (III). The spectra in water presented the contribution of both adducts, TEMPOL and TEMPON, due to subsequent reactions between OH radicals and the TEMPOL adduct (IV). The hyperfine coupling parameters for TEMPOL and TEMPON are given in the captions of Figure 2. Both the linewidth (Δ*H*_pp_) of the signals and the associated hyperfine coupling constant (aN) can be used to differentiate between TEMPOL and TEMPON [8].

Apart from the mechanism shown in III and IV, OH radicals are produced during the photosensitization of RB and PPIX via the production of O_2_^−^ and H_2_O_2_ [10,17]. In our investigations, the irradiation of RB resulted in higher OH radical production than PPIX (Figure 2B,C). For PPIX, a small production of OH radicals was observed at advanced reaction times, exceeding 30 min of irradiation. It is essential to account for the different contributions in the observed signals to accurately determine the production of ^1^O_2_. Figure 2A also includes a spectrum produced during the irradiation of PPIX in ethanol, which did not present a contribution of TEMPON signals. In this case, the OH radicals produced either via I or through other triplet state decay mechanisms probably reacted with the solvent (*k*_EtOH__+__OH_ = 2 × 10^9^ M^−1^ s^−1^), allowing for a cleaner detection of the TEMPOL adduct.


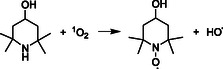
 (III)


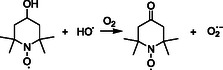
 (IV)

The dissolved oxygen (D.O.) concentrations were monitored in parallel control experiments in larger volumes that allowed the insertion of an oxygen probe, as described in the Supporting Information (Figure S3). These experiments aimed to attribute the observed TEMPOL signal to the formation of ^1^O_2_ via reactions with dissolved oxygen. The D.O. was linearly depleted up to 30 min, and 30 ± 3 μM of D.O. were consumed in experiments in the presence of 10 μM PPIX + 20 mM TEMP‐OH. In the absence of TEMP‐OH, the D.O. consumption was 23 μM. The lower D.O. consumption can be partially attributed to the absence of parallel reactions with TEMPOL (IV), but also due to PPIX aggregation effects. Since significant aggregation was observed at pH 7.5 in the absence of TEMP‐OH, lower D.O. consumption was expected. Therefore, the observed TEMPOL signals can be attributed to the ^1^O_2_ formed via the energy transfer between the excited triplet state of PPIX* and the dissolved ground‐state triplet oxygen (I).

The kinetics of TEMPOL formation, as shown in Figure 2B,C, were then used to estimate values for *I*_EPR_ using the following expressions



(1)

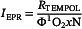






(2)

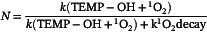




*R*_TEMPOL_ = initial rates of TEMPOL formation estimated from the slopes of the plot TEMPOL concentration over time presented in Figure 2B,C.

Φ^1^O_2__=_ quantum yield for singlet oxygen formation in water, equals 0.75 for RB and varies between 0.54 and 0.77 for PPIX depending on the experimental conditions.

*N* = probability that singlet oxygen reacts with TEMP‐OH before its decay in water [37].


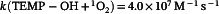


The value of Φ^1^O_2_ for PPIX calculations was 0.6, as this value was estimated using a Hg–Xe lamp and a pH of 7.4 [34], similar to the experimental conditions. Furthermore, the use of 0.6 as Φ^1^O_2_ for PPIX results in an estimated I_EPR_ closer to that estimated with RB, suggesting that this represents the correct Φ^1^O_2_ in the system. The choice of Φ^1^O_2_ can significantly affect the estimated *I*_EPR_ values, and variations of 60% can be observed when shifting the Φ^1^O_2_ from 0.54 to 0.77 under the investigated experimental conditions. Accordingly, *I*_EPR_ is in the range (1.3 ± 0.2) × 10^−8^ ≤ *I*_EPR_ ≤ (1.6 ± 0.3) × 10^−8^ mol photons L^−1^ s^−1^. Because environmental solar irradiation is key for initiating photochemistry in ambient samples, the irradiation of PPIX and RB solutions was then repeated under solar irradiation during clear sky days in the solar peak hour, as described in the methods section. The registered irradiance during the experiments varied from 500 to 600 W m^−2^. The determined *I*_SUN_ was (4.7 ± 0.4) × 10^−9^ mol photons L^−1^ s^−1^. The kinetics of TEMPOL evolution during solar irradiation experiments are shown in Figure 4A.

### 3.2 Application: Photochemical Production of ROS in SML Samples

For the determination of the photochemical production of ROS in SML samples, the EPR probe CMH was selected after several attempts to characterize the samples using the spin‐trapping agent 5,5‐dimethyl‐1‐pyrroline‐N‐oxide (DMPO). In complex matrices such as the SML samples, the chosen spin‐trapping agent or probe must compete with other matrix components for the radicals; therefore, it should be used in excess compared to the matrix components. The signal of the generated adduct must also be stable in the matrix and have a half‐life long enough to be detected in EPR experiments. Several compounds present in ocean samples were identified as sinks for ROS, especially organic matter and metals, such as Fe, Cu, and Mn [23]. Furthermore, in photo‐assisted experiments, the degradation of the probe upon irradiation must be considered, and corrections should be made to the signals if adducts form during the probe photolysis.

**FIGURE 3:**
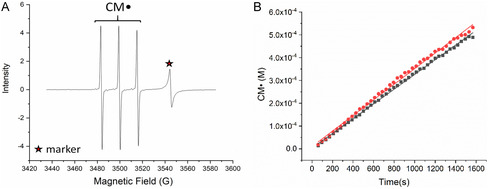
(A) Spectra of the formed CM• during irradiation of SML samples. (B) Linear profile obtained during the irradiation of SML samples. The black and red lines represent repetitions of the same SML sample.

Superoxide radicals (O_2_^−^) are long known to be one of the main ROS formed in ocean samples, with steady‐state concentrations reaching picomolar to nanomolar in several marine environments [24, 25]. The CMH probe is a known O_2_^−^ radical probe due to the relatively high rate constant of the reaction between CMH and O_2_^−^ radical, 1.2 × 10^4^ M^−1^ s^−1^ [28]. In contrast, other commonly used nitroxide spin‐trapping agents, such as DMPO, present very low rate constants with O_2_^−^, in the order of 0.8–50 M^−1^ s^−1^ for DMPO + O_2_^−^ [38]. Therefore, in complex matrices such as SML samples, the identification of O_2_^−^ radical in photo‐assisted experiments requires a large excess of the nitroxide, which in turn increases its photolysis rate. In this regard, the presence of O_2_^−^ radical can be readily inferred using CMH. Here, the rate for the uncatalyzed dismutation of O_2_^−^ in the SML samples at pH 8.5 is estimated as 1.8 × 10^4^ M^−1^ s^−1^ [39], which is in the same order of magnitude as the reaction between CMH and O_2_^−^. Therefore, the contribution of H_2_O_2_ formation should not be relevant under our experimental conditions, considering the excess of CMH (1 mM). The oxidation of CMH produces an aminoxyl radical (here represented by CM•) with a characteristic triplet EPR signal resulting from the hyperfine coupling between the unpaired electron and the nitrogen nucleus (aN) of the CM• structure (Figure 3A). The chemical mechanism for the formation of the radical is given in V [40]. Since CMH also reacts with other ROS and one‐electron oxidizing species, such as metal ions, with rate constants in the order of 10^4^ M^−1^ s^−1^ [28], the increase in the response of CMH in complex samples must be carefully analyzed. The formed CM• can also react with one‐electron reducers formed during the irradiation of CDOM, or act as a quencher of triplet states to regenerate the hydroxylamine [41].


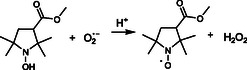
 (V)

The formation of CM• via reaction between CMH and O_2_^−^ radical is accompanied by the formation of H_2_O_2_. Although the CMH probe does not appreciably react with H_2_O_2_ [27], it can react with OH radicals coming from H_2_O_2_ decomposition via photolysis or Fenton‐like reactions. Due to the small produced H_2_O_2_ concentrations, the final rates of CM• formation (*R*_CM•_) are not expected to be influenced by these parallel reactions. Control experiments in the presence of H_2_O_2_ and kinetics aspects of the system were investigated to demonstrate the relevance of V in the production of OH radicals, and are described in the Supporting Information text S5.

**FIGURE 4:**
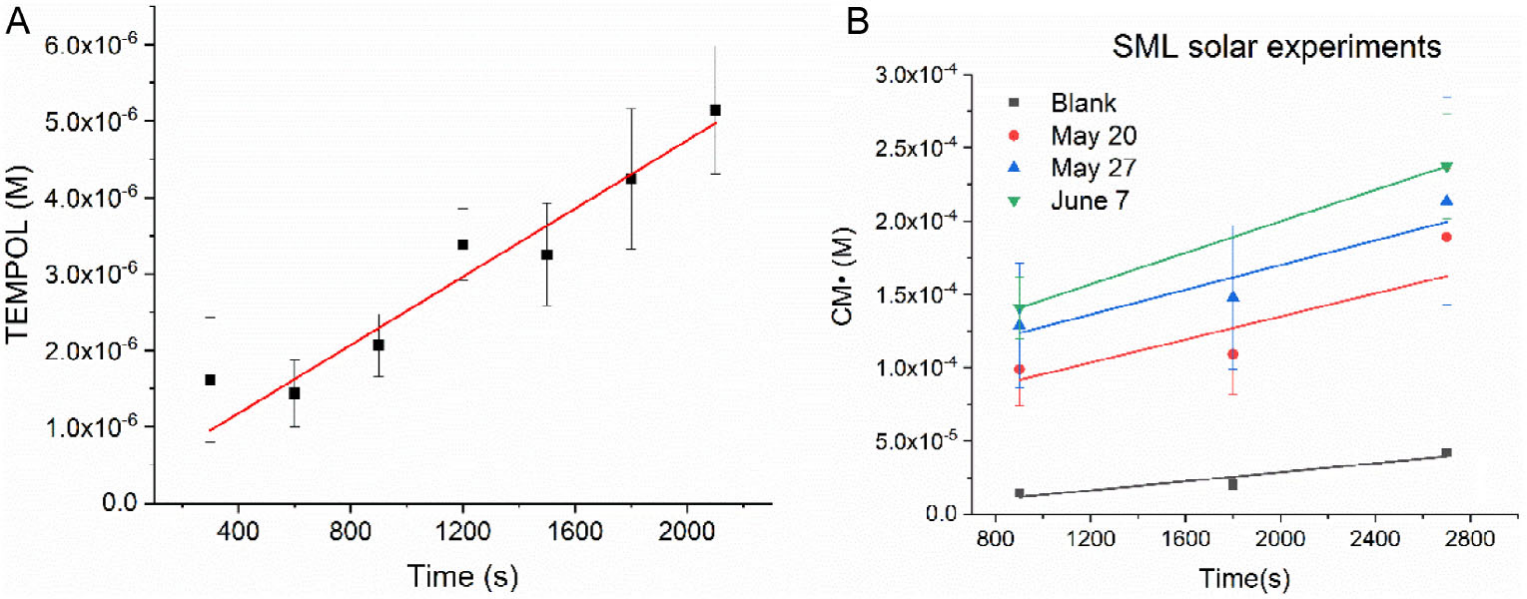
(A) TEMPOL formation during PPIX irradiation under solar peak hours. (B) Profiles of CM• formation during the exposure of SML samples to solar irradiation. Experiments were conducted in duplicates.

In the irradiation of SML samples, a linear formation of CM• was observed, indicating that the formed radicals are stable and can accumulate under the investigated conditions. Rates of CM• formation (*R*_CM•_) were used to compare different samples (Figure 3B). Three SML samples, collected on May 20, May 27, and June 7, were selected and exposed to solar irradiation simultaneously with the actinometer solutions used for the determination of *I*_SUN_ (Figure 4A,B). The formation of CM• under solar irradiation also presented linear trends. A blank of CMH solution was also exposed to irradiation, and the slopes of CM• formation were corrected with the blank values. As shown in Table 2, the results of the in situ EPR experiments can be compared with those conducted under solar irradiation, provided a correction is made using *I*_EPR_ and *I*_SUN_ as factors. The samples exposed to solar irradiation appear to be less photoactive than those exposed to the in situ irradiation with the lamp system, which reflects the lower *I*_SUN_. Since the solar spectrum is similar to the emission spectrum of an arc Xe‐lamp system, this simple correction, considering the differences in photon flux, is adequate to compare the rates of ROS formation and photochemically induced redox reactions in both experiments. This indicates that visible‐light‐driven photochemical processes predominate in the photochemical activity of SML samples, emphasizing the importance of such investigations.

**TABLE 2:** Rates of CM• formation in solar (*R*_CM• SUN_) and in situ EPR (*R*_CM• EPR_) experiments.^a^

*R*_CM•_ Sun, M s^−1^	*R*_CM•_ EPR, M s^−1^	Corrected *R*_CM•_ M s^−1^
(2.4 ± 0.5) × 10^−8^	(8.2 ± 0.5) × 10^−8^	(2.7 ± 0.4) × 10^−8^
(2.7 ± 0.4) × 10^−8^	(1.3 ± 0.1) × 10^−7^	(4.4 ± 0.6) × 10^−8^
(3.8 ± 0.5) × 10^−8^	(2.0 ± 0.8) × 10^−7^	(6.8 ± 0.9) × 10^−8^

a Corrections of *R*_CM•_ were done in *R*_CM•_ values measured in EPR in situ experiments using the relation (*I*_SUN_/*I*_EPR_ * *R*_CM•EPR_).

In the irradiation of SML samples, a linear formation of CM• was observed, indicating that the formed radicals are stable and can accumulate under the investigated conditions. Rates of CM• formation (*R*_CM•_) were used to compare different samples (Figure 3B). Three SML samples, collected on May 20, May 27, and June 7, were selected and exposed to solar irradiation simultaneously with the actinometer solutions used for the determination of *I*_SUN_ (Figure 4A,B). The formation of CM• under solar irradiation also presented linear trends. A blank of CMH solution was also exposed to irradiation, and the slopes of CM• formation were corrected with the blank values. As shown in Table 2, the results of the in situ EPR experiments can be compared with those conducted under solar irradiation, provided a correction is made using *I*_EPR_ and *I*_SUN_ as factors. The samples exposed to solar irradiation appear to be less photoactive than those exposed to the in situ irradiation with the lamp system, which reflects the lower *I*_SUN_. Since the solar spectrum is similar to the emission spectrum of an arc Xe‐lamp system, this correction, considering the differences in photon flux, is adequate to compare the rates of ROS formation and photochemically induced redox reactions in both experiments. This indicates that visible‐light‐driven photochemical processes predominate in the photochemical activity of SML samples, emphasizing the importance of such investigations.

Subsequently, in situ EPR irradiation was performed on 25 SML samples, and the results were corrected for equivalent solar exposure, resulting in an average sunlight‐induced oxidant formation rate under solar peak hours and 0% cloud coverage of (3.8 ± 0.5) 10^−8^ M s^−1^ (Table S1). Additional information on the samples, their composition, and concentrations of specific organic components can be found elsewhere [31]. These results reflect the maximum photochemical production of ROS and oxidants expected for the samples in days of clear skies in the springtime. Since O_2_^−^ radicals are considered one of the primary ROS formed in the photochemistry of SML samples, and considering the absence of signals in the presence of DMPO, the observed *R*_CM•_ can be partially attributed to O_2_^−^ radical formation. The monitored rates are considerably higher than previously measured rates of photochemical O_2_^−^ production in seawater samples, which range from < 1 to 2000 nM/h [42, 43, 44, 45]. Those measurements are based on the detection of superoxide and on its dismutation to H_2_O_2_. The measurement of the rates of O_2_^−^ formation is debatable, with some authors pointing to an oxidative sink for O_2_^−^ arriving from the production of phenoxy radical from DOM, which exceeds the rates of dismutation, not considered in most investigations [41, 46].

The SML samples contain significantly more DOM, which can lead to increased ROS production. Dissolved carbon is typically measured in SML samples at concentrations ranging from 100 to 1000 μM, usually rising during postbloom phases or due to anthropogenic influence [19, 21, 22, 36]. In the samples investigated here, DOC values range from 300 to 700 μM [31]. Additionally, the SML samples were collected during a mesocosm experiment with waters from the German Bight, an area that receives water from major German rivers. Reports indicate higher ROS production in estuary regions, exceeding 60 nM min^−1^ [47]. Thus, a greater ROS production likely occurs upon irradiation of the SML samples. Other processes, like metal redox chemistry, may also have contributed to the final observed *R*_CM•_ values, as discussed in our recent publication [31]. The investigated samples contained Fe and Cu in concentrations as high as 0.5 and 1.8 μM [31]. These metals can participate in cycles with CMH and act as sinks for O_2_^−^. Although soluble Fe and Cu ions present high rate constants with O_2_^−^ [39], a large fraction of the metals found in SML samples are probably in insoluble mineral forms or bound to organic ligands, making it difficult to estimate their real contribution to the observed *R*_CM•_ values.

Using the proposed actinometer solutions for direct EPR analysis of singlet oxygen formation, the in situ irradiation of samples can be corrected for the expected solar flux, and values closer to realistic scenarios can be obtained. Here, we applied the characterized system to the irradiation of SML samples, considering the natural relevance of these systems to the ocean composition and the aerosol emissions. These experiments provide direct evidence of the strong photochemical activity in SML samples. While the applied probe cannot resolve contributions from individual radicals, the absence of signals with conventional nitroxide traps strongly suggests that O_2_^−^ radical chemistry is an important pathway in the SML under natural irradiation.

## 4 Conclusion

A method to correct in situ EPR (*I*_EPR_) experiments for solar light exposure using chemical actinometers was investigated. Both Protoporphyrin IX (PPIX) and RB yielded similar *I*_EPR_ in the range (1.3 ± 0.2) × 10^−8^ ≤ *I*_EPR_ ≤ (1.6 ± 0.3) × 10^−8^. TEMP‐OH protonation interfered at pH < 2, while TEMP‐OH stabilized PPIX against aggregation. An in situ EPR probing method using CMH was developed to measure the contribution of O_2_^⋅‐^ and other one‐electron redox reactions formed in the irradiation of SML samples. SML samples showed a sunlight‐induced oxidant formation rate of (3.8 ± 0.5) 10^−8^ M s^−1^_._ Importantly, such investigations related to the SML had not been previously performed until the time of writing, and to the best of the authors’ knowledge. These findings highlight the potential of this approach to combine laboratory spectroscopy with real‐world photochemistry, providing a robust tool to quantify radical production in complex environmental systems.

## Supporting Information

Additional supporting information can be found online in the Supporting Information section. **Supporting** **Fig.** **S1:** Lamp emission spectrum adapted from the lamp manufacturer‘s catalog, contrasted to the measured irradiance and the cut‐off considering the glass capillary used in in situ EPR experiments. **Supporting** **Fig.** **S2:** Photobleaching effect in PPIX solutions pH 7.5 in the absence of TEMP‐OH. **Supporting** **Fig.** **S3:** Profiles of dissolved oxygen consumption in PPIX solutions pH 7.5 in the presence and absence of TEMP‐OH. **Supporting** **Fig.** **S4:** Example of solar irradiance measurements achieved during the days on which solar irradiation was performed for the calculation of photon flux and irradiation of SML samples. **Supporting** **Fig.** **S5:** A) Plots of rates of OH radical production (R_OH_) as a function of H_2_O_2_ concentrations, and B) Logarithm plot of rates of OH radical production (R_OH_) as a function of H_2_O_2_ concentrations Considering the following mechanism, and the sum of SR2 and SR3 given by SR4. **Supporting** **Table** **S1:** Data resulting from the irradiation of SML samples, and information on the sample collection date and time.

## Author Contributions

**Daniele Scheres Firak**: conceptualization (lead), data curation (lead), formal analysis (lead), investigation (lead), writing – original draft (lead). **Thomas Schaefer**: project administration (lead), writing – review & editing (supporting). **Bochao Yang**: formal analysis (supporting), investigation (supporting). **Olenka Jibaja Valderrama**: investigation (supporting), writing – review & editing (supporting). **Manuela van Pinxteren**: project administration (lead), writing – review & editing (supporting). **Hartmut Herrmann**: funding acquisition (lead), project administration (lead), resources (lead), writing – review & editing (lead).

## Conflicts of Interest

The authors declare no conflicts of interest.

## Supporting information

Supplementary Material
