# Supplementary material for: Coupling Aqueous Phase Chemical Actinometry with EPR Spectroscopy: An Approach for Probing Photochemical Processes at the Air–Sea Interface
Source: Chemphyschem. 2026 Feb 10;27(3):e202500734. doi: 10.1002/cphc.202500734 (PMC12890004; doi:10.1002/cphc.202500734)
Supplement: Supplementary file 1 — Supplementary Material [file CPHC-27-e202500734-s001.pdf]

Coupling Chemical Actinometry with EPR Spectroscopy: An Approach for Probing Photochemical Processes at the Air-Sea Interface

Daniele Scheres Firak, Thomas Schaefer, Bochao Yang, Olenka Jibaja Valderrama, Manuela van Pinxteren, Hartmut Herrmann*

Atmospheric Chemistry Department (ACD), Leibniz Institute for Tropospheric Research (TROPOS), Permoserstraße 15, 04318 Leipzig, Germany

Submitted to

ChemPhysChem

---

*Phone: ++49341 2717 7024. Fax: ++ 49341 2717 99 7024.

*Corresponding author e-mail: herrmann@tropos.de

S1 - Spectral characteristics of the lamp used in in-situ EPR experiments

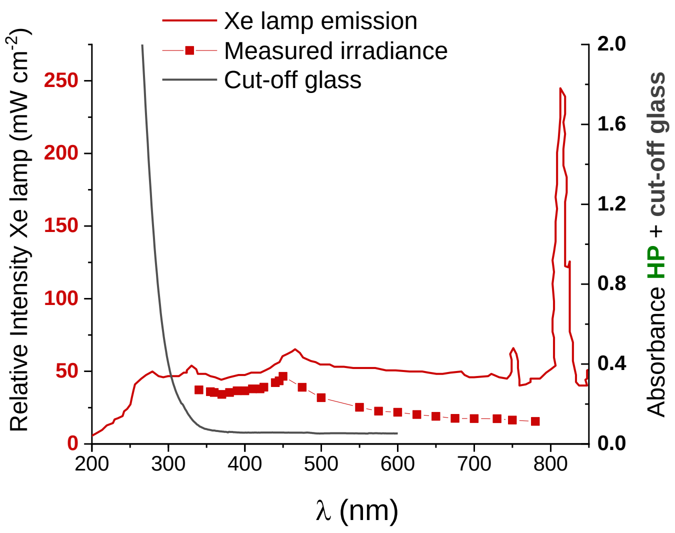

Figure S1: Lamp emission spectrum adapted from the lamp manufacturer's catalog, contrasted to the measured irradiance and the cut-off considering the glass capillary used in in-situ EPR experiments.

S2 - Photobleaching of Protoporphyrin IX (PPIX) solutions

The photobleaching experiments were prepared in 2 mL solutions, added to 5 mL glass flasks positioned in the direction of the light beam emanating from the optical fiber setup. The solutions were removed from the irradiation at predetermined times and transferred to a 5 cm path cuvette for reading in the UV-VIS spectrometer.

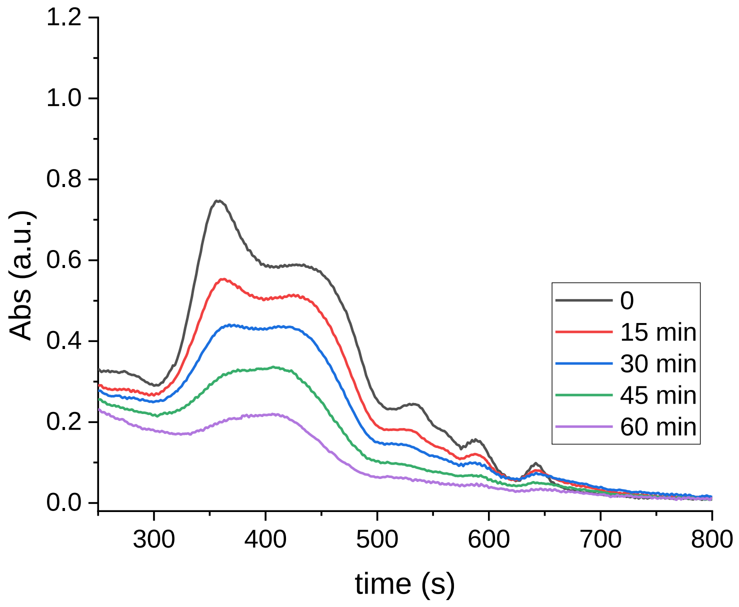

Figure S2: Photobleaching effect in PPIX solutions pH 7.5 in the absence of TEMP-OH

S3 - Monitoring of dissolved oxygen (D.O.) concentrations in the irradiation of Protoporphyrin IX (PPIX)

Solutions were prepared in a 5 mL glass flask sealed with a rubber cap, allowing for an oxygen probe (NEOFOX FOSPOR, Ocean Optics) to be inserted into the solutions. A syringe was used to add the reactants. The oxygen levels were initially measured in the PPIX solutions, and the initial D.O. concentrations were registered. The flasks were then positioned in the direction of a light beam emanating from the optical fiber setup used for the in situ EPR experiments. Although the same lamp system was used, the reaction vessel and the final volume of the solution were modified to permit the monitoring of the D.O. levels. Irradiations were done in the absence of the spin-trapping agent 4-Hydroxy-2,2,6,6-tetramethyl-piperidin (TEMP-OH) and in the presence of 20 mM aqueous TEMP-OH solutions. A linear decrease in D.O. concentrations was observed up to 30 minutes of irradiation, and the rate at later times appears to be lowered. The change in the rate of D.O. consumption might be caused by the photobleaching of PPIX.

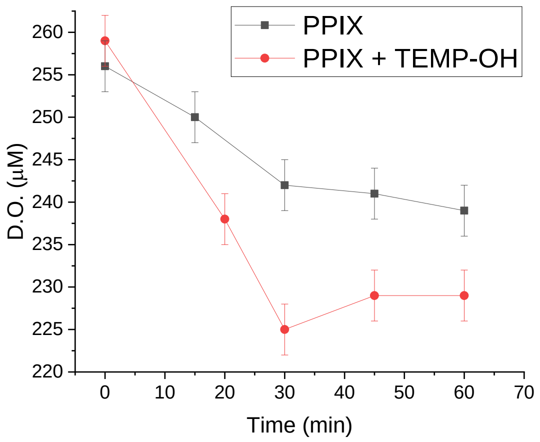

Figure S3: Profiles of dissolved oxygen consumption in PPIX solutions pH 7.5 in the presence and absence of TEMP-OH

S4 - Solar irradiance measurements

The solar irradiance was simultaneously measured using a silicon photodiode pyranometer (Model: ML-020VM), and the data were provided by the Remote Sensing Department of the Leibniz Institute for Tropospheric Research (TROPOS). The readings were checked daily to determine the optimal time and dates for completing the solar tests.

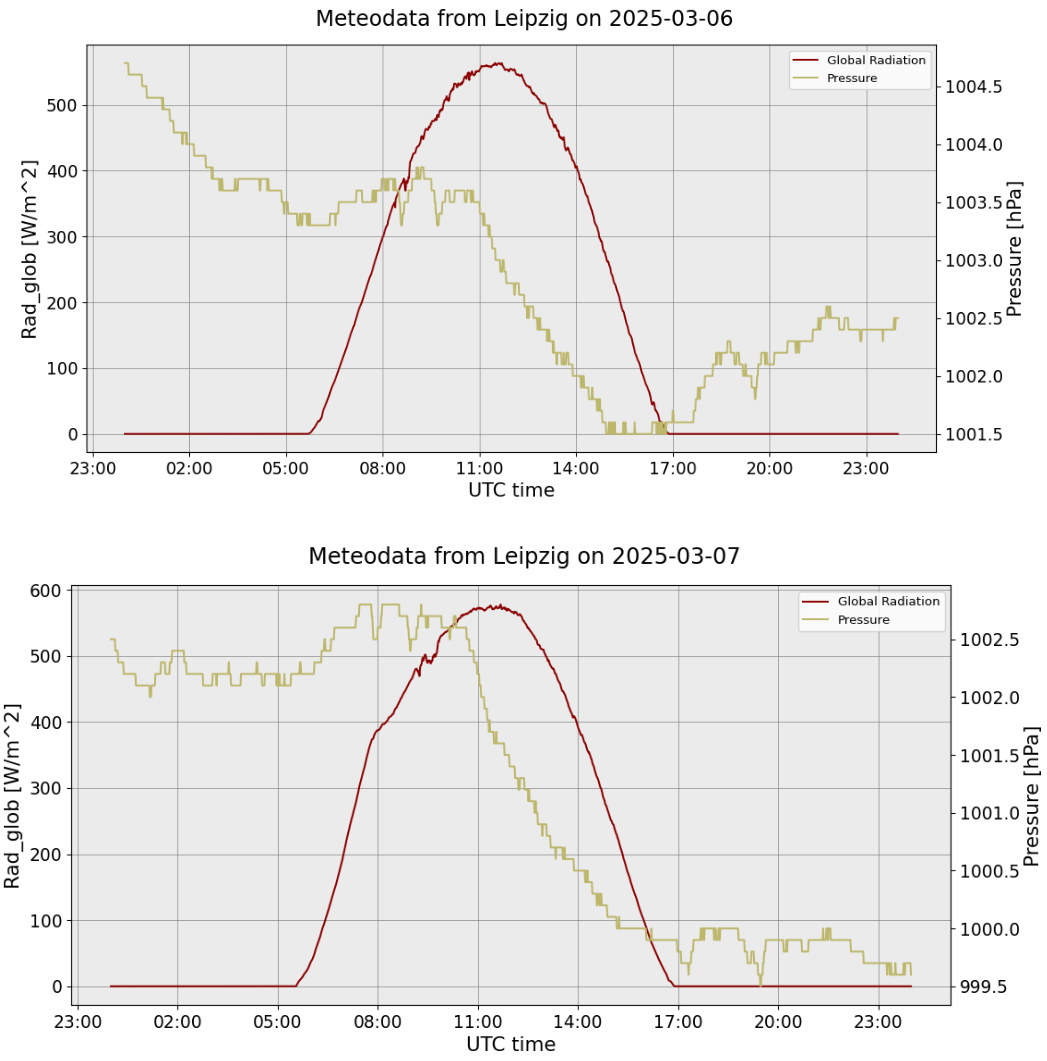

Figure S4: Example of solar irradiance measurements achieved during the days on which solar irradiation was performed for the calculation of photon flux and irradiation of SML samples

S5 - Control experiments in the presence of H_2O_2

To verify the influence of parallel formation of H_2O_2 during the probing of O_2^- radicals described by SR1, control experiments were performed at various H_2O_2 concentrations irradiated in the presence of 1-hydroxy-3-methoxycarbonyl -2,2,5,5-tetramethylpyrrolidine, CMH. Experiments of H_2O_2 photolysis were performed in water and in spiked SML samples. The range of chosen H_2O_2 concentrations was based on the final expected radical yields in SML samples after 30 minutes of irradiation (around 1×10^{-4} M). The rates of OH radical production (R_{OH}) were plotted as a function of H_2O_2 concentration to estimate the observed rate constants for the formation of HO radicals in each matrix (Figure S5 A). The rates at $[H_2O_2]_0$ correspond to the observed rates in the absence of H_2O_2, for both SML and water matrix. In water, these rates correspond to the photolysis of CMH, while in SML the rates correspond to the formation of O_2^- radicals + HO radicals. As seen in Figure S5 B, the reaction order in H_2O_2 is only equal to unity in water samples. In the SML samples, the formation of CM• arises from other matrix components, and the consumption of H_2O_2 occurs through different mechanisms. Importantly, the increase in H_2O_2 concentrations has less impact on the rate of CM• formation in SML samples than it has in water.

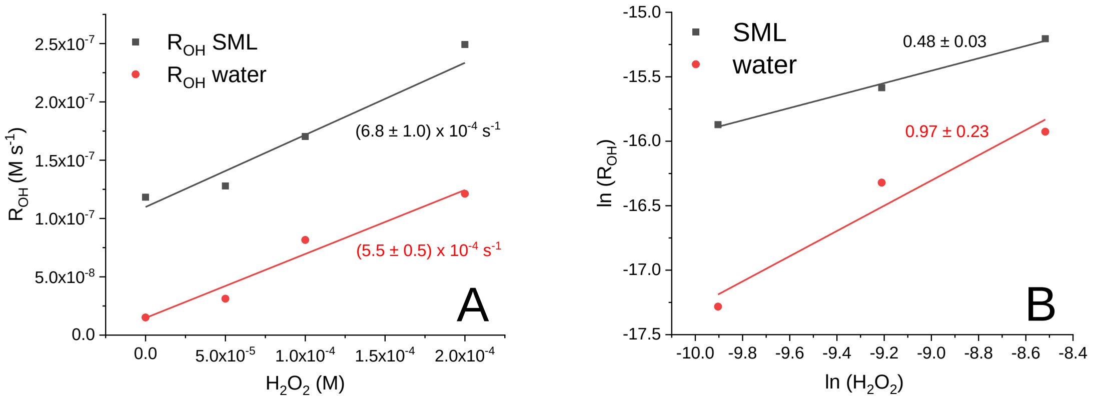

Figure S5: A) Plots of rates of OH radical production (R_{OH}) as a function of H_2O_2 concentrations, and B) Logarithm plot of rates of OH radical production (R_{OH}) as a function of H_2O_2 concentrations

Considering the following mechanism, and the sum of SR2 and SR3 given by SR4.:

$$CMH + O_2^{\bullet -} \xrightarrow{H^+} CM + H_2O_2 \quad \text{SR1}$$

$$\frac{1}{2} H_2O_2 \xrightarrow{h\nu} OH \quad \text{SR2}$$

$$OH + CMH \rightarrow CM\bullet \quad \text{SR3}$$

$$H_2O_2 \ + CMH \rightarrow \ CM \bullet \qquad \text{SR4}$$

The increase in CM• is followed in the EPR experiments, and SR1 and SR2 are consecutive reactions. The rate constant in k_{SR3}>>k_{SR2}; therefore, the observed rate k_{obs} extracted from the slope of R_{OH} vs. H_2O_2 (Figure S5A) corresponds to the SR2 rate.

The concentration of H_2O_2 is described by:

$$\frac{dH_2O_2}{dt} = k_{SR1}[CMH][O_2^{\bullet -}] - \frac{1}{2}k_{SR2}[H_2O_2] \qquad \text{SR5}$$

The rate of H_2O_2 production described by SR1 can be experimentally inferred from the experiments of SML irradiation in EPR experiments, considering R_{source} = k_{SR1}[CMH][O_2^-]. Considering R_{source} and applying steady-state conditions to SR5:

$$[H_2O_2]_{ss} = 2\,x\,\frac{R_{source}}{k_{SR2}}$$

If R_{source} = 1.2×10^{-7} M s^{-1} (average of experimental results of SML irradiation), and k_{SR2} is given by the slope of Figure S5A as 6.8×10^{-4} s^{-1}, $[H_2O_2]_{ss}$ is 2.8 $\times10^{-4}$ M. The steady state is reached after 1/kSR2, 24 minutes (1400 s). Therefore, the H_2O_2 formed in SR1 accumulates during SML irradiation, being slowly converted into CM•. Its concentration should not affect the estimated rates of O_2^- radical formation, which are obtained from the slopes of CM• formation in short irradiation times.

S6 – Additional information regarding the used SML samples

Samples were collected during a mesocosm experiment conducted at Sea-sURface Facility (SURF), at ICBM, Wilhelmshaven (Germany), between May 18th and June 16th, 2023. Samples were daily collected into pre-cleaned PTFE bottles, which were kept frozen until subsequent transportation to the final destination laboratory, where they were defrosted and distributed in smaller vials used for the EPR analysis.

Table S1. Data resulting from the irradiation of SML samples, and information on the sample collection date and time

SML sample	*Date*	*Local time*		*$R_{CM•}$ average*	*Corrected RCM•*
1	5/16/2023	6:51	Morning	$(1.0 \pm 0.2) \times 10^{-7}$	$3.3 \pm 0.7) \times 10^{-8}$
2	5/17/2023	16:12	Evening	$(2.3 \pm 0.5) \times 10^{-8}$	$7.5 \pm 0.9) \times 10^{-9}$
3	5/18/2023	6:50	Morning	$(1.1 \pm 0.1) \times 10^{-7}$	$3.6 \pm 0.1) \times 10^{-8}$
4	5/19/2023	16:06	Evening	$(1.4 \pm 0.3) \times 10^{-7}$	$4.4 \pm 0.9) \times 10^{-8}$
5	5/20/2023	6:45	Morning	$(8.2 \pm 0.5) \times 10^{-8}$	$2.6 \pm 0.1) \times 10^{-8}$
6	5/21/2023	15:55	Evening	$1.6 \pm 0.1) \times 10^{-7}$	$5.0 \pm 0.4) \times 10^{-8}$
7	5/22/2023	6:27	Morning	$1.4 \pm 0.1) \times 10^{-7}$	$4.6 \pm 0.4) \times 10^{-8}$
8	5/23/2023	15:16	Evening	$1.5 \pm 0.2) \times 10^{-7}$	$4.9 \pm 0.5) \times 10^{-8}$
9	5/26/2023	6:03	Morning	$1.2 \pm 0.2) \times 10^{-7}$	$3.9 \pm 0.7) \times 10^{-8}$
10	5/27/2023	15:35	Evening	$1.3 \pm 0.1) \times 10^{-7}$	$4.3 \pm 0.3) \times 10^{-8}$
11	5/28/2023	7:10	Morning	$1.0 \pm 0.1) \times 10^{-7}$	$3.4 \pm 0.2) \times 10^{-8}$
12	5/29/2023	15:20	Evening	$1.3 \pm 0.4) \times 10^{-7}$	$4.1 \pm 0.9) \times 10^{-8}$
13	5/31/2023	15:13	Evening	$9.2 \pm 0.1) \times 10^{-8}$	$3.0 \pm 0.1) \times 10^{-8}$
14	6/2/2023	15:07	Evening	$1.0 \pm 0.3) \times 10^{-7}$	$3.4 \pm 0.9) \times 10^{-8}$
15	6/3/2023	6:15	Morning	$1.3 \pm 0.1) \times 10^{-7}$	$4.3 \pm 0.1) \times 10^{-8}$
16	6/4/2023			$1.5 \pm 0.1) \times 10^{-7}$	$4.7 \pm 0.1) \times 10^{-8}$
17	6/5/2023	6:01	Morning	$1.2 \pm 0.1) \times 10^{-7}$	$3.8 \pm 0.5) \times 10^{-8}$
18	6/7/2023	5:30	Morning	$2.1 \pm 0.8) \times 10^{-7}$	$6.6 \pm 0.9) \times 10^{-8}$
19	6/8/2023	14:59	Evening	$5.5 \pm 0.1) \times 10^{-8}$	$1.8 \pm 0.1) \times 10^{-8}$
20	6/10/2023	15:15	Evening	$1.0 \pm 0.1) \times 10^{-7}$	$3.2 \pm 0.1) \times 10^{-8}$
21	6/11/2023	5:30	Morning	$1.8 \pm 0.2) \times 10^{-7}$	$5.8 \pm 0.6) \times 10^{-8}$
22	6/12/2023	15:30	Evening	$7.8 \pm 0.1) \times 10^{-8}$	$2.5 \pm 0.1) \times 10^{-8}$
23	6/13/2023	5:15	Morning	$1.4 \pm 0.1) \times 10^{-7}$	$4.5 \pm 0.1) \times 10^{-8}$
24	6/14/2023	15:15	Evening	$1.2 \pm 0.1) \times 10^{-7}$	$3.9 \pm 0.2) \times 10^{-8}$
25	6/15/2023	5:27	Morning	$5.9 \pm 0.2) \times 10^{-8}$	$1.9 \pm 0.6) \times 10^{-8}$
